# Water Cure

**Published:** 1846-10

**Authors:** 


*Water Cure.*—The Northampton (Massachusetts) Courier states that a Water Cure Establishment is about going into operation near that village, under the direction of an "intelligent and respectable colored gentleman," and that six of the wealthy citizens of the place generously contributed the sum of $3,000 to provide suitable buildings, &c. We wonder if six wealthy citizens could readily be found who would contribute a like sum for purposes of medical education! What glorious results in behalf of medical Science and humanity would follow, if a tithe of the enormous sums thrown away on the various forms of quackery were to be appropriated for those objects!

